# Health professionals` attitudes on smoking and cessation policies in forensic psychiatric hospitals

**DOI:** 10.1192/j.eurpsy.2026.11641

**Published:** 2026-07-31

**Authors:** H. Ryynänen, P. Sandström, H. Anthoni, S. Eskelinen, J. Niemi, E. Kivilinna, P. Korhonen, N. Tusa, T. Vasankari

**Affiliations:** 1 Finnish Lung Health Association; 2 Psychiatry, University of Helsinki and Helsinki University Hospital, Helsinki; 3 Vanha Vaasa Hospital, Vaasa; 4 Niuvanniemi Hospital, Kuopio; 5 Respiratory diseases and allergology, Turku University, Turku, Finland

## Abstract

**Introduction:**

Smoking is highly prevalent among patients with severe mental illnesses. Even though smoking cessation is known to improve both physical and mental health, smoking remains highly prevalent in Finnish psychiatric hospitals, while local policies and practices often fail to place sufficient emphasis on this. This study was conducted as a part of the Joint Action Prevent Non-Communicable Diseases -project (JA PreventNCD).

**Objectives:**

The aim of the study was to explore mental health personnel’s attitudes on the challenges and opportunities related to current smoking policies, and cessation practices, in two forensic psychiatric hospitals in Finland, which are the only state mental hospitals in the country.

**Methods:**

Data was collected from psychiatry personnel (health professionals) with an online survey during February and March 2025. Questions (open, multiple choice, numeric rating scale 1-10) included self-evaluation of attitudes, smoking cessation skills, smoking policies, and opinion on smoke/nicotine free psychiatric hospitals.

**Results:**

In total, 163 Health professionals answered the survey (response rate 25 %). Respondents rated the importance of cessation work on the numeric scale at 7.1, and skills in cessation support at 5.9 (image 1). Most respondents reported bringing up the use of nicotine products with their patients during their hospitalization quite regularly or always (89 %), but e.g. use of smoking dependency evaluation tests was uncommon (11 %). Main cessation methods used were individual counselling (68 %) and use of nicotine replacement therapy-products and/or prescription medicines (85 %) (image 2). While 77% saw smoke-free psychiatric hospitals as feasible in the future, only 53% believed that completely nicotine-free hospitals would be possible (image 3).

**Image 1:**
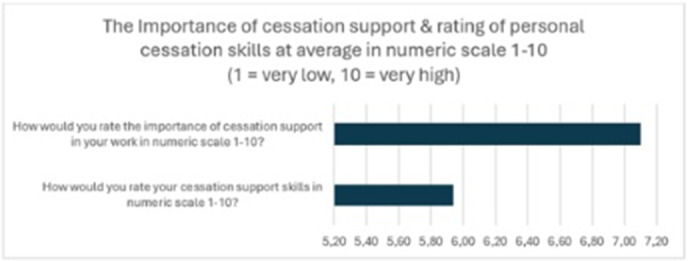

**Image 2:**
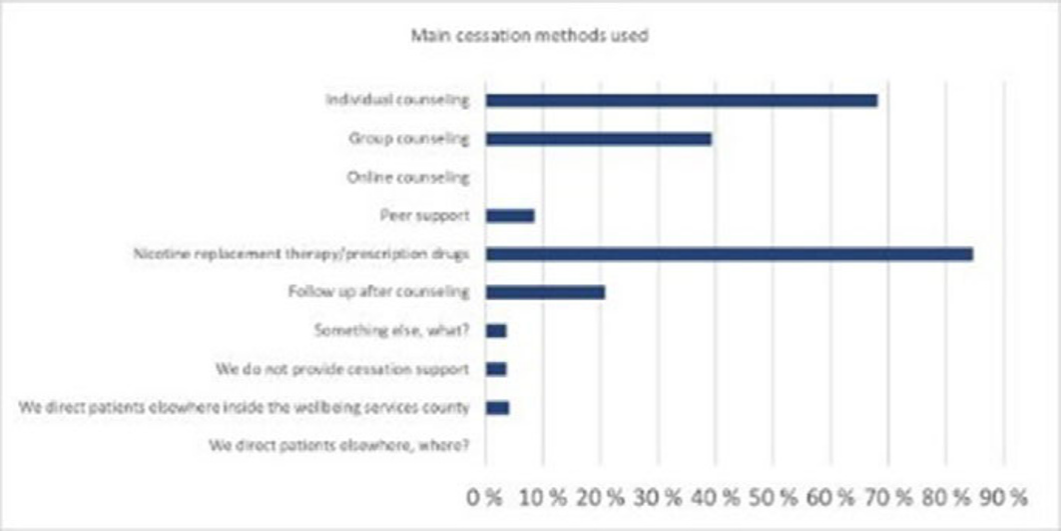
Image 2: Long description.

**Image 3:**
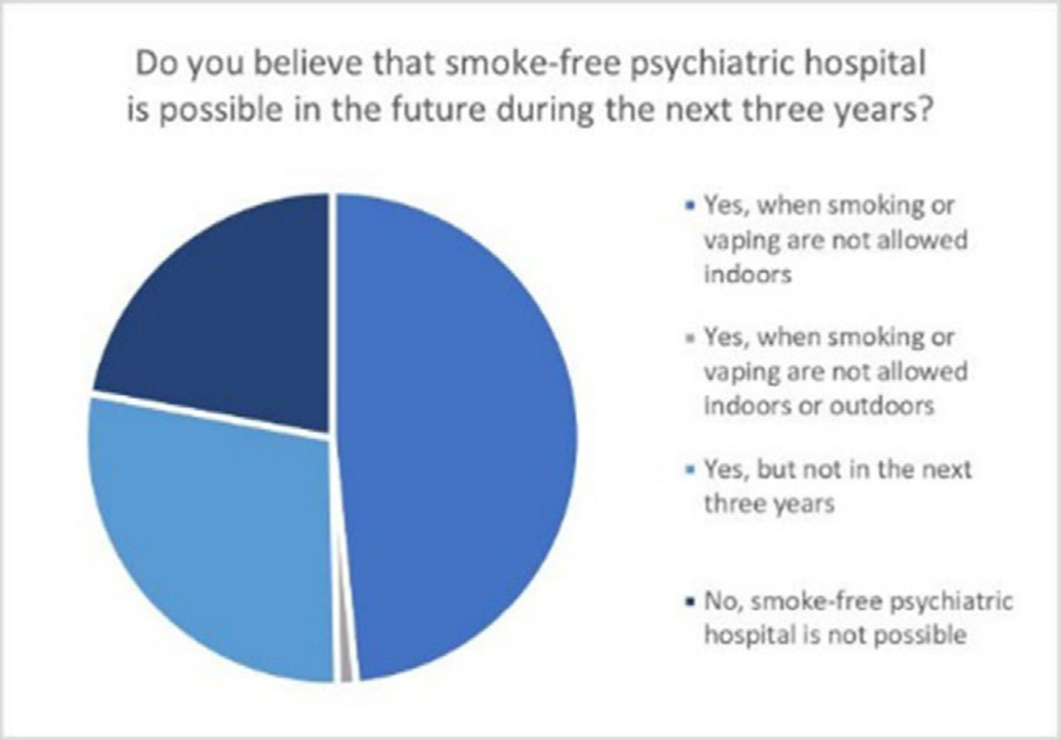
Image 3: Long description.

**Conclusions:**

Although psychiatric hospital staff recognize the importance of supporting patients in smoking cessation, they often lack the necessary expertise. To ensure structured and effective cessation practices, establishing clear policies, implementing standardized protocols, and providing comprehensive staff training is crucial. These preliminary results of this study have already been utilized in Vanha Vaasa Hospital, Finland, in enabling the transition into a completely interiorly smoke-free hospital.

**Disclosure of Interest:**

None Declared

## Long descriptions

### Image 2: Long description

The horizontal bar chart displays ten categories on the Y-axis with a percentage scale on the X-axis ranging from 0 percent to 90 percent in 10 percent increments.

* Individual counseling: approximately 68 percent.

* Group counseling: approximately 40 percent.

* Online counseling: 0 percent (no bar visible).

* Peer support: approximately 8 percent.

* Nicotine replacement therapy / prescription drugs: the highest value at approximately 85 percent.

* Follow up after counseling: approximately 21 percent.

* Something else, what?: approximately 4 percent.

* We do not provide cessation support: approximately 4 percent.

* We direct patients elsewhere inside the wellbeing services county: approximately 4 percent.

* We direct patients elsewhere, where?: 0 percent (no bar visible).

Navigate back to Image 2:.

### Image 3: Long description

The chart is titled Do you believe that smoke-free psychiatric hospital is possible in the future during the next three years? It is divided into four segments.

* The largest segment, starting from the 12 o'clock position and moving clockwise to approximately the 6 o'clock position, is medium blue. It corresponds to the legend item: Yes, when smoking or vaping are not allowed indoors.

* A very thin sliver at the bottom center is light gray. It corresponds to the legend item: Yes, when smoking or vaping are not allowed indoors or outdoors.

* The next segment, moving clockwise from the bottom to the 9 o'clock position, is light blue. It corresponds to the legend item: Yes, but not in the next three years.

* The final segment, completing the circle from the 9 o'clock position back to the top, is dark blue. It corresponds to the legend item: No, smoke-free psychiatric hospital is not possible.

Navigate back to Image 3:.

